# 2-Hydroxypropyl-beta-cyclodextrin Treatment Does Not Induce Atherosclerotic Lesion Regression in Western-Type Diet-Fed Apolipoprotein E Knockout Mice

**DOI:** 10.3390/biom12091205

**Published:** 2022-08-31

**Authors:** Olga S.C. Snip, Menno Hoekstra, Yiheng Zhang, Janine J. Geerling, Miranda Van Eck

**Affiliations:** 1 Division of BioTherapeutics, Leiden Academic Centre for Drug Research, Leiden University, 2333 CC Leiden, The Netherlands; 2 Division of Systems Pharmacology and Pharmacy, Leiden Academic Centre for Drug Research, Leiden University, 2333 CC Leiden, The Netherlands; 3 Pharmacy Leiden, Leiden, The Netherlands

**Keywords:** cyclodextrin, cholesterol efflux, macrophages, atherosclerosis regression, mouse model

## Abstract

2-Hydroxypropyl-beta-cyclodextrin (2HPβCD) is able to bind and solubilize unesterified cholesterol and may therefore be able to reverse the deposition of cholesterol in macrophages within the aortic vessel wall, a hallmark of atherosclerotic cardiovascular disease. However, conflicting results regarding the potential of 2HPβCD to induce regression of established atherosclerotic lesions have been described. In the current study, we therefore also investigated the ability of 2HPβCD to stimulate cholesterol removal from macrophage foam cells in vitro and induce the regression of established atherosclerotic lesions in apolipoprotein E knockout (APOE KO) mice. In vitro studies using murine thioglycollate-elicited peritoneal macrophages verified that 2HPβCD is able to induce cholesterol efflux from macrophages in an ATP-binding cassette transporter-independent manner. Switching Western-type-diet-fed APOE KO mice with established atherosclerotic lesions back to a chow diet was associated with a reduction in the hypercholesterolemia extent and an increase in the absolute lesion size and plaque collagen-to-macrophage ratio. Importantly, parallel subcutaneous administration of 2HPβCD was not able to prevent the diet-switch-associated lesion growth or induce atherosclerosis regression. Although in our hands, 2HPβCD does effectively stimulate cellular cholesterol efflux from macrophages, we do not consider it worthwhile to further pursue 2HPβCD as therapeutic moiety in the atherosclerosis regression context.

## 1. Introduction

Deposition of cholesterol in macrophages within arterial walls leading to lumen occlusion is a hallmark of atherosclerosis, the primary underlying cause of cardiovascular disease [1]. Given that hypercholesterolemia is a major risk factor for the development of macrophage foam cells and atherosclerotic lesions, cholesterol-lowering approaches—i.e., treatment with statins—are currently employed as the number one cardiovascular therapy. Unfortunately, the statin treatment-associated reduction in low-density lipoprotein (LDL)-cholesterol levels reduces the cardiovascular disease risk by maximally 25% [2]. Thus, there remains a clear need for alternative therapeutic approaches to tackle the residual cardiovascular risk.

Starch-derived chemically modified cyclic oligosaccharide 2-Hydroxypropyl-beta-cyclodextrin (2HPβCD) is able to bind and solubilize unesterified cholesterol [3]. As such, treatment with this compound may be a promising therapeutic strategy to relief macrophages from their excessive cholesterol load and reduce the atherosclerotic cardiovascular disease risk. In agreement, data published by Zimmer et al. in 2016 suggest that treatment with 2HPβCD beneficially impacts macrophage cholesterol handling and is able to induce regression of existing atherosclerotic lesions in hypercholesterolemic apolipoprotein E knockout (APOE KO) mice [4]. More specifically, Zimmer et al. have found that combining a switch from a pro-atherogenic cholesterol-enriched diet to a normal (low-fat) chow diet with 2HPβCD treatment leads to a reduction in total lesion area and a decrease in lesional cholesterol crystal content. Strikingly, a recent study by Kim et al. has detected a null effect of treatment with 2HPβCD in APOE KO mice on atherosclerosis outcome under diet-switch cholesterol lowering conditions [5]. The sizes of atherosclerotic lesions in the aortic root and the descending aorta were virtually identical in mice treated with 2HPβCD and the solvent control in the study of Kim et al., whilst they did find a reduction in plaque size after treatment with beta-cyclodextrin polymer [5]. Given that the reproducibility of findings is essential for a proper interpretation of study outcomes, in the current study we also investigated the ability of 2HPβCD to stimulate cholesterol removal from macrophage foam cells in vitro and induce the regression of established atherosclerotic lesions in APOE KO mice. Furthermore, we performed a detailed comparison of the overlap and differences between the (published) 2HPβCD-related atherosclerosis regression studies to potentially explain the differential study outcomes.

## 2. Materials and Methods

### 2.1. Cholesterol Efflux Assay

Female C57BL/6 wild-type mice and mice genetically lacking the ATP-binding cassette transporters ABCA1 and ABCG1 (A1/G1 DKO mice) were obtained from in-house breedings and injected intraperitoneally with 3% Brewer’s thioglycollate (Sigma, Steinheim, Germany). Five days later, mice were killed through cervical dislocation. Thioglycollate-elicited macrophages were harvested by lavage of the peritoneal cavity with 10 mL PBS, washed, and plated in 24 well plates in DMEM containing 1 g/L glucose, 25 mM HEPES, 1% L-glutamine, and 1% penicillin-streptomycin (Lonza, Geleen, The Netherlands) supplemented with 10% heat-inactivated fetal calf serum. Following overnight attachment to the culture plates, the peritoneal macrophages were loaded with 1 µCi/mL [1α,2α(n)-3H]-cholesterol (Perkin Elmer, Groningen, The Netherlands) and 0.003% non-labeled cholesterol (Sigma) in DMEM supplemented with 0.2% fatty acid free BSA (Sigma) for 24 h. Subsequently, the cells were washed with PBS and left to equilibrate for 1 h in DMEM with 0.2% fatty-acid-free BSA-containing DMEM. Thereafter, cholesterol efflux was initiated through providing cells with DMEM supplemented with either 2.5% mouse serum from C57BL/6 mice or 2HPβCD (Sigma, Steinheim, Germany) for 4 h. Radioactivity in the cells (lysed for 30 min in 0.1 M NaOH) and the medium was measured by scintillation counting (Beckman Coulter, Brea, CA, USA). The cholesterol efflux rate was calculated by dividing the amount of radioactivity present in the medium by the total radioactivity found in the medium and cell lysate.

### 2.2. Atherosclerosis Regression Study

Female APOE KO mice aged 9 ± 1 weeks, obtained from in-house breedings, were randomly allocated to either the baseline group, control group, or 2HPβCD treatment group. All three groups were fed a Western-type diet with 16% fat (15% cacao butter and 1% corn oil), 20% casein, and 0.25% cholesterol for 6 weeks, which was followed by the sacrifice of the baseline group (N = 15). The remaining mice were switched to a regular chow-diet (soy bean protein base) and subcutaneously injected every other day with either 2 g/kg body weight 2HPβCD (Sigma; N = 15) or a similar volume of its solvent control PBS (120–140 µL, depending on the mouse body weight; N = 15) for 4 weeks. Mice were anesthetized through subcutaneous injection with 100–150 µL of a mix of xylazine (70 mg/kg), ketamine (350 mg/kg), and atropine (1.8 mg/kg). Blood was collected retro-orbitally in EDTA-coated tubes and centrifuged for 10 min at 6000 rpm to obtain plasma samples that were stored at −20 °C until further use. Subsequently, the mice were perfused in situ with PBS (pressure 100 mmHg) via a cannula in the left ventricular apex. Hearts were collected and stored in 4% formalin. All mice were housed in climate-controlled stables in individually ventilated cages and exposed to a 12 h day/night cycle. During the full course of the experiment, mice had ad libitum access to food and water. Animal experiments were performed in accordance with the ARRIVE guidelines and approved by the Dutch Central Commission for Animal experimentation (Centrale Commissie voor Dierproeven) according to the Dutch Law on laboratory animal experimentation and the EU Directive 2010/63/EU. All experimental protocols were approved by the Ethics Committee for Animal Experiments of Leiden University.

### 2.3. Plasma Cholesterol Measurement

To determine the level of cholesterol in plasma samples, an enzymatic colorimetric assay was performed as described by Out et al. [6]. The absorbance was measured at 490 nm using a BioTek PowerWave HT microplate spectrophotometer. Precipath (Roche Diagnostics, Rotkreuz, Switzerland) was used as reference.

### 2.4. Histological Analysis of Atherosclerotic Lesions

Aortic roots were sectioned transversely at 10 µm intervals using a CM3050 S cryostat (Leica Ltd., Rotterdam, The Netherlands) by embedding the hearts in Tissue-Tek O.C.T. Compound (Sakura Finetek, Alphen aan den Rijn, NL). A Leica DMRE microscope and Leica Qwin Imaging software (Leica Ltd., Cambridge, UK) were used to obtain images. Atherosclerotic lesion areas, total vessel areas, and lesional lipid, macrophage, and collagen contents were quantified in a blinded manner. Neutral lipids were identified using a standard Oil red O staining (Sigma, Steinheim, Germany). Masson’s Trichrome (Sigma, Steinheim, Germany) was used to stain collagen fibers with hematoxylin as the counterstain. Macrophages were stained immunohistochemically using an anti-CD68 antibody. Cholesterol crystals were visualized under polarized light.

### 2.5. Hematological Analysis

Leukocyte, neutrophil, lymphocyte, and monocyte concentrations were routinely measured using an automated SYSMEX XT-2000iV Veterinary Heamatology analyzer (Sysmex Europe GMBH, Norderstedt, Germany) in eye blood collected at sacrifice. The neutrophil-to-lymphocyte ratio (NLR) was calculated for each individual sample by dividing the blood neutrophil concentration by the associated blood lymphocyte concentration.

### 2.6. Plasma Cytokine Analysis

Levels of the pro-inflammatory cytokines monocyte chemoattractant protein-1 (MCP-1), interleukin-6 (IL-6), and tumor necrosis factor-alpha (TNF-alpha) were measured in plasma samples that were diluted 2 times in Assay Diluent using the ELISA MAX^TM^ Standard (MCP-1 & IL-6) and DeLuxe (TNF-alpha) Set kits from BioLegend (San Diego, CA, USA) according to the manufacturer’s instructions.

### 2.7. Statistical Analysis

Statistical analysis was performed using Graphpad Instat software (San Diego, CA, USA). Normality testing of the experimental groups was performed using the method of Kolmogorov and Smirnov. Outliers were detected using a Grubb’s test. The significance of differences between groups was calculated using a *t*-test, or one or two-way analysis of variance (ANOVA) with the Bonferroni post-test, where appropriate. Probability values of *p* < 0.05 were considered significant.

## 3. Results

**Figure 1:**
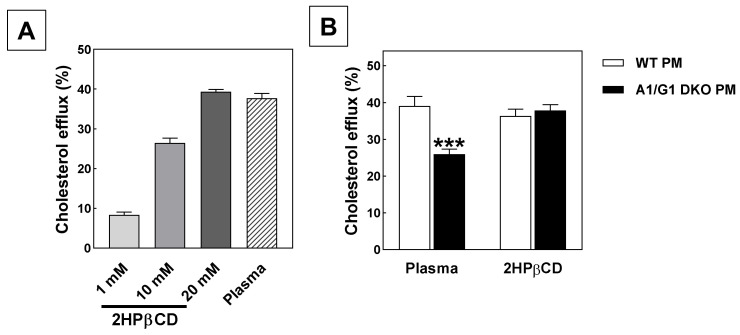
2HPβCD induces macrophage cholesterol efflux in an ABC transporter-independent manner. (**A**) Cholesterol efflux from thioglycollate-elicited peritoneal macrophages of C57BL/6 wild-type mice exposed to different concentrations of 2HPβCD or 2.5% mouse plasma (positive control) for 4 hours. (**B**) Cholesterol efflux from thioglycollate-elicited peritoneal macrophages from C57BL/6 wild-type (WT PM) and ABCA1/ABCG1 double knockout (A1/G1 DKO PM) mice exposed to 2.5% mouse plasma or 10 mM 2HPβCD for 4 h. Data represent the means + SEM of 6 wells. *** *p* < 0.001 versus WT.

To verify that the ability of 2HPβCD to solubilize cholesterol translates into the effective removal of cholesterol from lipid-laden macrophages, we determined the effect of 2HPβCD treatment on the macrophage cholesterol efflux in vitro. A dose-dependent increase in the efflux of cholesterol from thioglycollate-elicited peritoneal macrophages was detected, with cholesterol efflux rates at 20 mM 2HPβCD being comparable to those of the positive cholesterol acceptor control (2.5% pooled plasma from C57BL/6 wild-type mice; Figure 1A).

Zimmer et al. have previously demonstrated that the atherosclerosis regression-inducing effect of 2HPβCD is not dependent on the presence of the cholesterol efflux mediators ATP-binding cassette transporter A1 (ABCA1) and ABCG1 in macrophages [4]. As can be appreciated from Figure 1B, the plasma-stimulated efflux of cholesterol from peritoneal macrophages genetically lacking both ABCA1 and ABCG1 (−34%; *p* < 0.001) was significantly reduced as compared to wild-type controls, which is in line with the concept that nascent and mature high-density lipoproteins in plasma require a functional interaction with ABCA1 and ABCG1, respectively, to facilitate cholesterol efflux [7]. Macrophage ABCA1/ABCG1 deficiency did, however, not impact the ability of 10 mM 2HPβCD to stimulate cholesterol efflux (Figure 1B). In agreement with the suggestion from Zimmer et al. that 2HPβCD activates the conversion of cholesterol to 27-hydroxycholesterol for subsequent passive efflux [4], 2HPβCD thus activates macrophage cholesterol removal in a manner not dependent on active transport by ABCA1/ABCG1 in our in vitro setting.

**Figure 2:**
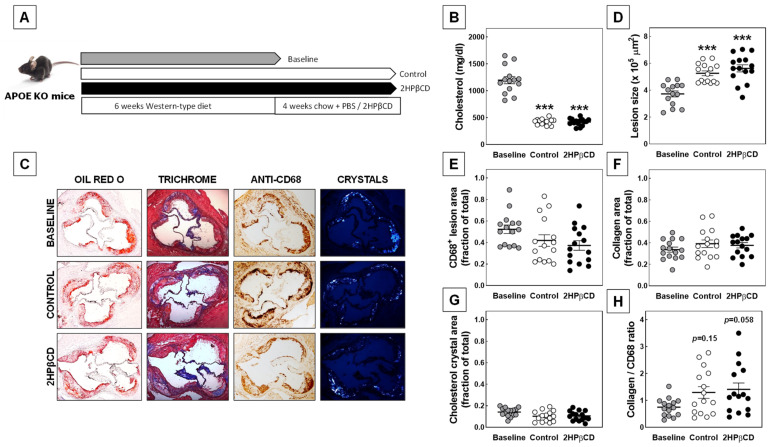
2HPβCD treatment fails to induce regression of established atherosclerotic lesions in APOE KO mice. (**A**) Schematic overview of the three experimental groups of female mice used in our regression study. Plasma total cholesterol levels (**B**), aortic root atherosclerotic lesion sizes (**D**), the lesional relative CD68^+^ macrophage content (**E**), collagen content (**F**), cholesterol crystal content (**G**), and collagen-to-macrophage ratio (**H**) in female APOE KO mice that were fed a Western-type diet for 6 weeks (Baseline) or subsequently also fed a chow diet for 4 weeks and treated with PBS (Control) or 2 g/kg 2HPβCD. Data are presented as individual data points and the respective group means ± SEM. *** *p* < 0.001 versus Baseline. Representative images of plaque stainings for neutral lipids (Oil red O), collagen (Trichrome), macrophages (anti-CD68), and cholesterol crystals are displayed in panel (**C**).

To study the potential of 2HPβCD to induce atherosclerosis regression in vivo, three groups of age-matched female APOE KO mice were first fed a Western-type diet enriched in cholesterol and fat for 6 weeks to speed up the development of atherosclerotic lesions. Subsequently, one group of mice was sacrificed to serve as baseline reference group. The remaining two experimental groups were switched back to the normal (low fat; no cholesterol added) chow diet for 4 weeks to reverse the exacerbated hypercholesterolemia and were injected subcutaneously every other day with a dose of 2 g/kg body weight of 2HPβCD or a similar amount of the solvent control to evaluate the potential of the established lesions to regress. Please refer to Figure 2A for a graphical overview of the study setup.

APOE KO mice exhibited severe hypercholesterolemia with average plasma total cholesterol concentrations of >1000 mg/dL after 6 weeks of Western-type diet feeding (Figure 2B). As a result, atherosclerotic lesions were readily detected in the aortic root of the Western-type-diet-fed baseline group through staining sections for neutral lipid and collagen deposits using Oil red O and Masson’s Trichrome, respectively (Figure 2C). Quantification of the different plaque parameters revealed that the baseline group of mice exhibited aortic root atherosclerotic lesions of 3.7 ± 0.2 × 10^5^ µm^2^ (Figure 2D) that primarily consisted of CD68^+^ macrophages (~50% of total plaque area; Figure 2E) and collagen deposits (~30% of total lesion area; Figure 2F). Furthermore, a substantial amount of cholesterol crystals could be found in the lesions at baseline (see the representative images in Figure 2C and the quantification in Figure 2G).

Switching the mice back to the normal chow diet was associated with the anticipated decrease in plasma total cholesterol levels (Figure 2B). The parallel 2HPβCD treatment did not affect the diet-switch-associated drop in the hypercholesterolemia extent. Both groups of APOE KO mice exhibited plasma cholesterol levels of ~420 mg/dL after 4 weeks of chow diet feeding. Notably, despite the marked reduction in the hypercholesterolemia extent, significantly larger atherosclerotic lesions were found as compared to those detected at baseline in the control-treated chow-diet-fed mice (5.3 ± 0.2 × 10^5^ µm^2^; one way ANOVA *p* < 0.001; Figure 2D). The apparent increase in lesion size coincided with a relative increase in lesional collagen content and a decrease in the percentage of lesion area attributable to macrophages and cholesterol crystals (Figure 2E,F). As a result, the diet switch was associated with a marked increase in the plaque collagen-to-macrophage ratio (+74%; *t*-test: *p* < 0.05; One-way ANOVA *p* = 0.15; Figure 2H), a measure of plaque stability.

Importantly, as also evident from the representative images in Figure 2C, average lesion sizes were virtually identical in 2HPβCD-treated (5.6 ± 0.3 × 10^5^ µm^2^) and PBS-treated (5.3 ± 0.2 × 10^5^ µm^2^) chow-diet-fed mice (*p* > 0.05). In further support of an overall null effect of 2HPβCD treatment on atherosclerosis outcome in our experimental setting, lesional relative macrophage, collagen, and cholesterol crystal contents as well as the plaque collagen-to-macrophage ratio were not significantly different between the two groups of chow-diet-fed mice (Figure 2E–H).

**Figure 3:**
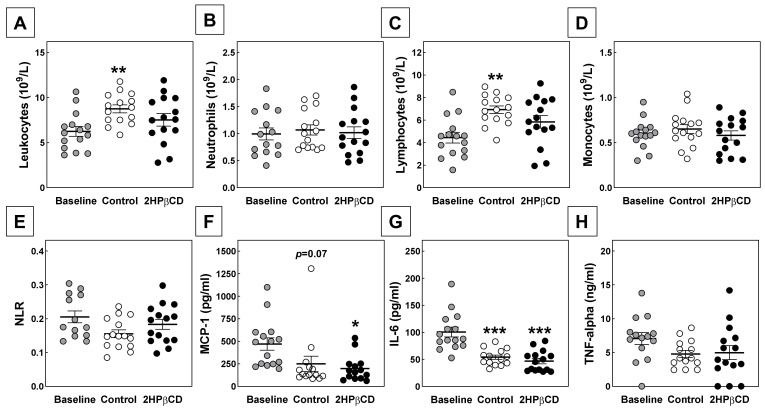
2HPβCD treatment does not change the systemic inflammatory state. Blood total leukocyte (**A**), neutrophil (**B**), lymphocyte (**C**), and monocyte (**D**) counts, the calculated NLR (**E**), and plasma levels of the pro-inflammatory cytokines MCP-1 (**F**), IL-6 (**G**), and TNF-alpha (**H**) in female APOE KO mice that were fed a Western-type diet for 6 weeks (Baseline) or subsequently also fed a chow diet for 4 weeks and treated with PBS (Control) or 2 g/kg 2HPβCD. Data are presented as individual data points and the respective group means ± SEM. * *p*<0.05, ** *p* < 0.01, *** *p* < 0.001 versus Baseline.

Studies by Houben et al. have suggested that 2HPβCD, in addition to its cholesterol-depleting effect, can stimulate the production of pro-inflammatory cytokines by macrophages [8]. Since inflammation is a key process in the pathogenesis of atherosclerosis, we evaluated whether the inability of 2HPβCD treatment to induce regression of atherosclerotic lesions was paralleled by potentially important changes in the inflammatory state. For this purpose, we performed routine hematological analysis on orbital blood specimens from the different groups of mice collected during sacrifice. Importantly, although the switch from a Western-type diet towards a chow diet was associated with significant changes in the hematological profile, no relevant additional effect was detected of the parallel 2HPβCD treatment. Chow-diet-fed mice as compared to Western-type-diet-fed mice exhibited higher blood concentrations of leukocytes (Figure 3A), which is possibly related to the fact that the chow-diet-fed mice had received multiple subcutaneous injections. The relatively high white blood cell counts in chow-diet-fed mice could solely be attributed to an increase the number of lymphocytes and was independent of the treatment (Figure 3B).

Blood neutrophil and monocyte counts were not affected by the diet switch and not significantly different between 2HPβCD-treated and PBS-treated mice (Figure 3C,D). As a result, also no significant effect of the diet switch or 2HPβCD treatment on the NLR—a marker for subclinical inflammation—was detected; 0.18 ± 0.01 for 2HPβCD-treated mice versus 0.16 ± 0.01 for PBS-treated mice and 0.21 ± 0.02 for Western-type-diet-fed controls, respectively (Figure 3E).

Analysis of pro-inflammatory cytokine levels in plasma specimens further verified that 2HPβCD treatment did not affect the systemic inflammatory state in our experimental setting. The switch from the Western-type diet to the chow diet was associated with a trend towards a decrease in plasma levels of MCP-1 (−47%; One-way ANOVA *p* = 0.07) as well as a significant reduction in plasma levels of IL-6 (−46%; *p* < 0.001) in control-treated mice, while the diet-switch-associated 32% decrease in average plasma TNF-alpha levels did not reach significance. Plasma levels of MCP-1 and IL-6 were also markedly decreased in chow-diet-fed 2HPβCD-treated mice as compared to the Western-type-diet-fed baseline group −58% (*p* < 0.05) and −53% (*p* < 0.001), respectively). However, no difference was detected in plasma levels of the three cytokines between 2HPβCD-treated mice and PBS-treated mice.

## 4. Discussion

With the current study we aimed to provide a definite answer as to whether or not 2HPβCD is able to induce atherosclerosis regression. A switch to a chow diet induced a reduction in the hypercholesterolemia extent and increased the absolute lesion size and plaque collagen-to-macrophage ratio in Western-type-diet-fed APOE KO mice. However, parallel subcutaneous administration of 2HPβCD was not able to prevent the diet-switch-associated lesion growth or induce atherosclerosis regression in our experimental setting. Although we were able to verify—in our in vitro setting—the hypothesis of Zimmer et al. that the presence of the macrophage cholesterol transporters ABCA1 and ABCG1 is not required for 2HPβCD-mediated cholesterol efflux [4], our in vivo atherosclerosis findings thus do not replicate those of Zimmer et al. (2HPβCD-mediated regression induction) [4] and rather provide additional support for the conclusion of Kim et al. that treatment with 2HPβCD is ineffective in inducing atherosclerosis regression (null effect of 2HPβCD) [5].

The three study designs were, in general, quite similar with an initial period of cholesterol-rich atherogenic diet feeding of APOE KO mice and a subsequent switch to a chow diet with parallel subcutaneous injections with either 2HPβCD or solvent control (see Table 1 for a side-by-side study setup and outcome comparison). However, potentially relevant differences between the utilized experimental approaches have been uncovered. As can be appreciated from Table 1, the composition of the diet that was provided to the mice to stimulate the development of atherosclerotic lesions was significantly different between the three studies. We used a relatively mild atherogenic Western-type diet with only 0.25% (*w*/*w*) of added cholesterol, whilst the more atherogenic high-fat diets of Zimmer et al. and Kim et al. contained 1.25% (*w*/*w*) cholesterol. Kim et al. also supplemented their atherogenic diet with 0.5% cholic acid, which is anticipated to even further increase the hypercholesterolemia and atherosclerosis extent as compared to diets devoid of this bile acid [9]. Kim et al. did not include a baseline group and we thus do not know which plasma cholesterol levels their mice actually obtained during the initial atherogenic diet challenge. However, in all three studies, plasma cholesterol levels were reduced to similar levels in both treatment groups during the following chow-diet feeding period. It thus appears that the type and the time (varying between 6 and 12 weeks in these three studies) of atherogenic diet feeding does not impact on the ability of plasma cholesterol levels to return to normal values for chow-diet-fed APOE knockout mice (400–500 mg/dL). A contribution of this specific parameter to the differences in final atherosclerosis outcome after the diet switch can thus highly likely be eliminated.

Differences have previously been found in the susceptibility of male and female APOE KO mice to atherosclerosis, with a higher predisposition in females [10,11]. This can also explain why the respective atherosclerotic lesion extents were highly similar in our study and that of Kim et al., despite the markedly different combinations of the sex of the mice used and time of atherogenic diet feeding (6 weeks mild atherogenic diet feeding in female mice versus 12 weeks extreme atherogenic diet feeding in male mice, respectively). Given that these two specific studies both found a null effect of 2HPβCD treatment on atherosclerosis outcome, we assume that sex is also not a variable contributing to the differential study outcomes. However, it is important to acknowledge that the sex of the mice used by Zimmer al. has not been reported. As such, it is difficult to perform a good pair-wise comparison between Zimmer’s atherosclerosis (regression) findings and those of the other two studies. An additional complicating factor in this context is the fact that the lesion quantification methods used are also different. We and Kim et al. used the generally accepted method to measure absolute aortic root lesion size areas (ín µm^2^), while Zimmer et al. presented their lesion size data as the percentage of total vessel wall area. As evident from Table 1, we also corrected the absolute lesion size for the total vessel area and found markedly lower percentages in all our experimental groups as compared to Zimmer et al. (25–30% versus 32–59%, respectively). On the basis of this finding, one can hypothesize that the larger baseline lesions detected in the study of Zimmer et al. were more prone to regress in response to the combined diet switch and 2HPβCD treatment. However, we feel that additional experimental confirmation for this hypothesis is warranted. It remains unclear to us from their materials and methods section what Zimmer et al. actually considered the vessel wall area. Does this measurement include the valves, media, and/or adventitia? Regardless, we do not consider adjusting the lesion size to aortic root size justified, because the aortic root of APOE KO mice is known to feature a consistent remodeling response that is closely related to local lesion development [12]. In support of this, the diet-switch-associated increase in absolute atherosclerotic lesion size observed in the current study was also associated with enlargement of the aortic root, ultimately leading to an overall unchanged lesion area / vessel wall area ratio. In our opinion, vessel wall area corrected values may therefore not properly reflect the actual atherosclerotic lesion extent.

In conclusion, in our hands, 2HPβCD does effectively stimulate cellular cholesterol efflux from macrophages in vitro, but fails to induce regression of established atherosclerotic lesions induced by mild Western-type feeding in the aortic root of APOE KO mice. Since our in vivo findings corroborate those of Kim et al., we do not consider it worthwhile to further pursue 2HPβCD as therapeutic moiety in the atherosclerosis regression context. However, it is of interest that Kim et al. did find a beneficial effect on atherosclerosis outcome of treatment with beta-cyclodextrin polymer [5]. Confirmatory studies are therefore warranted on the regression-inducing potential of this specific cyclodextrin variant. In light of the difficulties we faced when comparing the different study designs and outcomes, we want to stress that it is of utmost importance to report the complex experimental setups of atherosclerosis regression studies in accordance with the ARRIVE (Animals in Research: Reporting In Vivo Experiments) guidelines and to adhere to standardized protocols for analyzing atherosclerotic lesion size [13,14].

## Figures and Tables

**Table 1:** Comparison of 2HPβCD-related atherosclerosis regression study setups and outcomes.

Parameter	Zimmer et al. [4]	Kim et al. [5]	Current Study
Mice	APOE KO on a C57BL/6J background (Charles River)	APOE KO on a C57BL/6J background (Jackson Laboratories)	APOE KO on a C57BL/6J background (Jackson Laboratories)
Number of mice per group	6–8	6	15
Sex	Unknown	Male	Female
Age at study initiation	12 weeks	10 weeks	8–10 weeks
Atherosclerosis induction	8 weeks cholesterol-rich diet containing 1.25% cholesterol, 21% fat, and 19.5% casein (Ssniff)	12 weeks Paigen’s high-fat diet, containing 1.25% cholesterol, 16% fat, and 0.5% cholic acid (Diet D12336; Research Diets)	6 weeks Western-type diet, containing 0.25% cholesterol and 16% fat (15% cacao butter and 1% corn oil), and 20% casein (Diet W; Special diets services)
Regression induction	4 weeks chow diet (unknown supplier)	4 weeks chow diet (unknown supplier)	4 weeks chow diet (special diets services)
Compound treatment	Subcutaneous injection with 2HPβCD (2 g/kg bodyweight; unkown supplier) or 0.9% NaCl as vehicle control twice a week	Subcutaneous injection with 2HPβCD (1 g/kg bodyweight; Cyclolab) or its solvent PBS as vehicle control twice a week	Subcutaneous injection with 2HPβCD (2 g/kg bodyweight; Sigma) or its solvent PBS as vehicle control every other day
Plasma total cholesterol levels	Baseline: 900 mg/dL Control: 500 mg/dL 2HPβCD: 350 mg/dL	Baseline: Unknown Control: 390 mg/dL 2HPβCD: 360 mg/dL	Baseline: 1198 mg/dL Control: 423 mg/dL 2HPβCD: 420 mg/dL
Aortic root lesion sizes	Baseline: 59% of total vessel area Control: 54% of total vessel area 2HPβCD: 32% of total vessel area	Baseline: Unknown Control: 35 × 10^4^ µm^2^ 2HPβCD: 32 × 10^4^ µm^2^	Baseline: 37 × 10^4^ µm^2^ (24.9% of total area) Control: 53 × 10^4^ µm^2^ (29.0% of total area) 2HPβCD: 56 × 10^4^ µm^2^ (29.3% of total area)

Average plasma total cholesterol levels and aortic root lesion sizes from the studies of Zimmer et al. and Kim et al. were estimated from the relevant bar graphs displayed in their respective publications.

## Data Availability

All data related to this study that are not already presented in this manuscript are available from the authors upon reasonable request.
